# Validation of the Naimigen questionnaire among the healthy population of Russia during the COVID-19 pandemic

**DOI:** 10.1192/j.eurpsy.2022.1288

**Published:** 2022-09-01

**Authors:** J. Koniukhovskaia, E. Pervichko, O. Mitina, O. Stepanova, I. Shishkova, E. Dorokhov, V. Petrenko

**Affiliations:** 1 Pirogov Russian National Research Medical University, Clinical Psychology Department, Moscow, Russian Federation; 2 Lomonosov Moscow State University, Psychology, Moscow, Russian Federation; 3 Ryazan State Medical University named after I.P. Pavlov, Faculty Of Clinical Psychology, Ryazan, Russian Federation

**Keywords:** Covid-19 pandemic, dysfunctional breathing, psychosomatics, Naimigen questionnaire

## Abstract

**Introduction:**

The Naimigen questionnaire (Van Dixhoorn, Duivenvoordent, 1985) was developed in the 1980s to assess the severity of hyperventilation syndrome, which causes respiratory alkalosis and, as a result, polysystemic functional symptoms. Later, this questionnaire was recommended for use in the diagnosis of dysfunctional breathing. The COVID-19 pandemic provokes anxiety as a stressful event and objectifies the respiratory function, which becomes a favorable ground for the growth of the prevalence of dysfunctional breathing in society.

**Objectives:**

To validate the Naimigen questionnaire in the context of the COVID-19 pandemic among the Russian-speaking population.

**Methods:**

The author’s socio-demographic questionnaire and the Naimigen Questionnaire (NQ) were used (Van Dixhoorn, Duivenvoordent, 1985). The study was conducted online from April 27 to December 28, 2020. It was attended by 1,362 people from all regions of Russia, including 1,153 women and 209 men aged 15 to 88 years (38.3 ±11.4).

**Results:**

The stable reliability of the Alpha-Kronbach coefficients (> 0.877) was revealed for all NQ points. To check the factor structure of the Naimigen questionnaire, we conducted an exploratory factor analysis using the direct Oblimin criterion, which, when explaining 57.3% of the total variance, allowed us to identify 4 factors: respiratory symptoms, paresthesia and gastrointestinal symptoms, tension, derealization.

**Conclusions:**

Checking the reliability and factor structure of the Naimigen questionnaire allows us to reasonably use this questionnaire on a Russian-language sample in the conditions of the COVID-19 pandemic. Disclosure: Research is supported by the Russian Science Foundation, project No. 21-18-00624.

**Disclosure:**

Research is supported by the Russian Science Foundation, project No. 21-18-00624.

